# Ethyl Pyruvate Pretreatment Attenuates Concanavalin A-Induced Autoimmune Hepatitis in Mice

**DOI:** 10.1371/journal.pone.0087977

**Published:** 2014-02-03

**Authors:** Miao Shen, Jie Lu, Ping Cheng, Chunlei Lin, Weiqi Dai, Fan Wang, Chengfen Wang, Yan Zhang, Kan Chen, Ling Xu, Yinqun Zhou, Chuanyong Guo

**Affiliations:** Department of Gastroenterology, Shanghai Tenth People’s Hospital, Tongji University of Medicine, People‘s Republic of China; University of Milano, Italy

## Abstract

**Pharmacological Relevance:**

Ethyl pyruvate (EP), a potent reactive oxygen species scavenger, has been reported to contribute to the inflammatory process. However, the protective effect of ethyl pyruvate on Concanavalin A (Con A)-induced autoimmune hepatitis have not been explored. Thus, the aims of this study are to investigate both the effects of ethyl pyruvate and its mechanism of protection on Con A-induced autoimmune hepatitis in mice.

**Materials and Methods:**

Acute autoimmune hepatitis was induced by Con A (20 mg/kg) in Balb/C mice; ethyl pyruvate (40 mg/kg and 80 mg/kg) was administrated 1h prior to the Con A injection. At 3h, 6h and 24h post Con A injection, histological grading, proinflammatory cytokine levels and nuclear factor kappa B (NF-κB) activity were determined.

**Results:**

Following Con A challenge, cytokines TNF-α, IL-2, IL-1β and IL-6 were expressed at 3h and 6h, and the level of HMGB1 significantly increased by 24h. Pretreatment with ethyl pyruvate ameliorated the pathological effects of Con A-induced autoimmune hepatitis and significantly decreased the levels of TNF-α, IL-2, IL-6 and IL-1β at 3h and 6h and the level of HMGB1 at 6h and 24h post injection. Ethyl pyruvate blocked the degradation of IκB α and IκB β and decreased the expression of NF-κB at 24h.

**Conclusion:**

Taken together, these results indicated that ethyl pyruvate protected against Con A-induced autoimmune hepatitis by decreasing both early (TNF-α, IL-2, IL-1β and IL-6) and late (HMGB1) cytokine expression in mice. The reduction of HMGB1 may correlate with the amelioration of NF-κB activity.

## Introduction

Hepatitis seriously threatens human health and daily life due to a high incidence of transmitted disease. There are several types of hepatitis, including viral hepatitis, autoimmune hepatitis, and alcoholic hepatitis. With an understanding of the basic mechanism surrounding hepatitis, several treatments have been used, but current, therapeutics contribute little to the cure of hepatitis. Therefore, more effective therapies need to be explored to provide new applications in the clinic.

Concanavalin A (Con A) has the ability to activate T cells to secrete cytokines, resulting specifically in liver injury serving as a T cell mitogen. Pathological studies show the infiltration and accumulation of large quantities of lymphocytes in liver parenchyma, mainly CD4^+^ T cells [1]. Therefore, Con A is well known as an inducer of T cell-mediated hepatitis, especially in the model of autoimmune hepatitis. In addition to the infiltration of effecter cells, mostly CD4^+^ T cells, kupffer cells, and natural killer T (NKT) cells, the secretion of proinflammatory cytokines such as IL-1β, IL-6, TNF-α and IFN-γ also play an important role in the early development of inflammation [2], [3], [4], [5], [6].

High-mobility group box 1 (HMGB1) is a highly conserved nuclear protein first purified from nuclei approximately 30 years ago [7]. HMGB1 is a non-histone protein and studies on function of it have shown a role in construction and stability of nucleosomes, regulation of gene transcription, and DNA repair [8]. The research of Wang et al. [9] revealed that HMGB1 can be released into the cytoplasm to induce inflammation. And HMGB1 has since attracted worldwide attention as a late cytokine. HMGB1 is widely found in the lymph tissue, liver, brain, spleen, kidney, and heart. HMGB1 can be either actively released by immune cells in response to stimulation by LPS, or passively released by damaged and necrotic cells. Once secreted into the cytoplasm, HMGB1 induces inflammation by engaging with multiple receptors, such as the Toll-like receptor-2 (TLR2), TLR4, TLR9, and the receptor for advanced glycation end products (RAGE) [10], [11], [12].

Recent studies have suggested that HMGB1 may be a mediator of inflammation, and has been implied in various diseases, such as ischemia reperfusion injury and atherosclerosis [13], [14]. Furthermore, HMGB1 has been implicated in acute liver injury [15] and Tong et al. [16] have reported that inhibition of HMGB1 activity alleviated liver injury in heatstroke. HMGB1 was also identified as an early mediator of injury and inflammation in hepatic ischemia-reperfusion injury, demonstrated by Watanabe et al. [17]. Blockade of HMGB1 by a neutralizing antibody inhibited proinflammatory cytokine production, NF-κB activity, and as a result, attenuated Con A-induced hepatitis in mice, as reported by Gong et al. [18]. In addition to neutralizing antibodies, other agents have also been shown to be protective in inflammatory diseases, partly through decreasing systemic HMGB1 accumulation, such as quercetin, green tea and curcumin [19], [20], [21]. Therefore, HMGB1 has the potential to become a therapeutic target in treating inflammatory diseases. Ethyl pyruvate (EP) is a stable lipophilic ester derivative of pyruvate with the structural formula, CH3COCOOCH2-CH2, and a molecular weight of 116.18 Da [22]. The exploration of ethyl pyruvate began with the study of the antioxidation of pyruvic acid. Pyruvic acid is the final product of the glycolytic cycle and the initiator of gluconeogenesis, and is the primary product in the process of energy metabolism. Pyruvic acid participates in the elimination of reactive oxygen species (ROS) to alleviate oxidative injury. However, the use of pyruvic acid as a therapeutic agent is limited by its aqueous instability. Ethyl pyruvate was first used as the succedaneum of pyruvic acid in animal experiments; it is more stable and less toxic compared to pyruvate acid. Ethyl pyruvate has now been used in various animal models [23]; for example, it has been shown to ameliorate acute pancreatitis and peritonitis [24]. Ethyl pyruvate also has protective effect after ischemia/reperfusion injury in heart, brain and kidney by reducing the release of HMGB1 [25]. Lu-Wen Wang et al. [26] demonstrated that ethyl pyruvate protects against experimental acute-onset chronic liver failure in rats. However, it is not known whether ethyl pyruvate attenuates Con A-induced hepatitis, an autoimmune disease model in Balb/c mice. In general, the mechanism of action of ethyl pyruvate has not been clearly determined.

In the present study, we probe the effects of ethyl pyruvate on Con A-induced autoimmune hepatitis and explore the mechanism of ethyl pyruvate action further.

## Materials and Methods

### 2.1 Reagents

Ethyl pyruvate and Con A were purchased from Sigma-Aldrich (USA). Enzyme-linked immunosorbent assay (ELISA) kits for IL-2, IL-6, TNF-α and IL-1β were purchased from R&D systems (Minneapolis, MN, USA). HMGB1 ELISA kit was purchased from Shino-Test Corporation (Oonodai, Kanagawa, Japan). Antibodies used in this study include HMGB1 (Epitomics, CA), IL-2 (Biolegend, CA), IL-6 (Proteintech, CA), TNF-α (Santa Cruz, CA), NF-κB (Proteintech, CA), IL-1β (Biolegend, CA), IκB α (Cell signal technology, CA) and IκB β (Cell signal technology, CA).

### 2.2 Animals

Male Balb/c mice (6–8 weeks old, 22±2 g) were purchased from Shanghai Laboratory Animal Co Ltd (SLAC, Shanghai, China). The mice were housed in an environment at a temperature of 25±2°C with an alternating 12h light and dark cycle; they were permitted free access to standard laboratory food and water. All animal experiments were approved by the Animal Care and Use Committee of Shanghai Tongji University.

### 2.3 Experiment #1: Con A Treatment

Con A was dissolved in normal saline solution at a concentration of 20 mg/kg according to the prior report [23]. 100 mice were randomly divided into two groups: group A was injected with saline solution through the tail vein and group B was injected with Con A at 20 mg/ml. 5 mice randomly-selected from group A and group B were killed at designed time points: 0h, 2h, 4h, 6h, 8h, 10h, 12h, 14h, 18h and 24h after Con A injection. Serum and liver tissue samples were then collected and frozen at −80°C until analyzed for cytokine levels and liver enzymes.

### 2.4 Experiment #2: Pre-treatment of Con A-treated Mice with Ethyl Pyruvate

Based on the levels of IL-2, IL-6, TNF-α, IL-1β and serum alanine transaminase (ALT) and aspartate transaminase (AST) observed in the first set of experiments, we set the time points at 3h, 6h and 24h. Next, we divided 72 mice randomly into four groups of 18 mice each.Each group received the following treatment.

Group I: normal control (n = 18); mice were injected in the tail vein with saline solution only.

Group II: model group (n = 18); mice were injected in the tail vein with 20 mg/kg Con A.

Group III: protected group (n = 18); mice were injected in the tail vein with ethyl pyruvate(40 mg/kg) 1h prior to Con A challenge.

Group IV: protected group (n = 18); mice were injected in the tail vein with ethyl pyruvate (80 mg/kg) 1h prior to Con A challenge.

Six mice from each group were randomly selected and were killed at time points 3h, 6h and 24h. All serum and liver tissue sample were collected and stored at −80°C.

### 2.5 Biochemical Analysis

#### 2.5.1 Serum aminotransferase assay

After blood collection, serum was separated by centrifugation at 2000 rpm at room temperature for 10 min. To detect the level of hepatocellular injury following Con A challenge, serum ALT and AST were measured by an automated chemistry analyzer (Olympus AU1000, Japan).

#### 2.5.2 Serum cytokine measurement

To assess the serum levels of IL-2, IL-1β, IL-6, TNF-α and HMGB1, ELISA kits were used according to the manufacturer’s instructions.

### 2.6 Histopathology

A portion of the liver tissue was preserved in 4% paraformaldehyde for at least 24 hours, and paraffin blocks were prepared according to the standard protocol [10]. Sections 3 µm thick were cut and stored at room temperature. The paraffin sections were then stained with hematoxylin and eosin (H&E) to observe the level of inflammation and tissue damage by light microscopy.

### 2.7 Immunohistochemistry

Prepared paraffin-embedded sections were dewaxed and rehydrated through a series of graded alcohols followed by heating in a baking oven at 60°C for 20 min. Antigen was recovered in citrate buffer incubated in a 95°C water-bath for 20 min and then endogenous peroxidase was blocked by incubating in 3%hydrogen peroxide for 20 min at 37°C. Membranes were ruptured with 0.2% triton at room temperature for 30 min and non-specific binding sites were blocked with 5% BSA at 37°C for 20 min followed by room temperature incubation for 10 min. The liver slices were then incubated overnight with rabbit anti-mouse HMGB1 (1∶500) and rabbit anti-mouse NF-κB (1∶50). On the second day, slices were incubated with secondary antibody (goat anti-rabbit) (Epitomics, CA) for 30 min at room temperature. The analysis of antibody binding was performed using a DAB kit. Afterwards, slides were counterstained with hematoxylin, dehydrated using graded ethanol and xylene, and mounted with Entellan. Slides were then observed by light microscopy. The assay was carried out by using Image-Pro Plus software 6.0 (Media Cybernetics, Silver Spring, MD, USA). The integrated optical density (IOD) of HMGB1 was calculated in our results. IOD is equal to the number of density (mean) multiply area, which can exactly indicate the amount of protein expressing in cytoplasm. And the rate of positive nucleus of NF-κB was also calculated using Image-Pro Plus 6.0. Three different fields of vision were random selected in one slide, and the IOD of them were acquired with Image-Pro Plus 6.0. We calculated the average of these three IOD. The same method was administrated in other two mice random selected from the same group. The above method was applied in all groups.

### 2.8 Immunofluorescence

Fresh liver tissue from mice was fixed in 4% paraformaldehyde for 1h on ice. Fixed liver tissue was washed with PBS for 5 min three times on ice. The liver tissue was then dehydrated in 30% sucrose (dissolved in PBS) overnight at 4°C. Liver tissue was infiltrated in OCT for 2 hours on day 2. The liver tissue was then frozen and stored at −80°C. Sections of 5 µm were cut with a freezing microtome and preserved at −20°C for preparation. Prepared sections were dried at room temperature for 5 min. OCT was then dissolved in PBS for 5 min, membranes were ruptured with 0.2% Triton at room temperature for 20 min, and non-specific antigen binding site was blocked by 5% BSA. Sections were then incubated with rabbit anti-mouse HMGB1 at 4°C overnight. Nuclear staining was performed by DAPI (1∶1000) after incubating with goat anti-rabbit antibody for 30 min on day 2. All sections were observed with a fluorescence microscope.

### 2.9 Western Blot Analysis

Liver tissues were recovered from −80°C storage and rapidly ground in liquid nitrogen and then lysed with RIPA lysis buffer and protease inhibitor. The protein concentration was detected using the BCA method. Equivalent amounts of total protein (120 µg) were boiled and subjected to sodium dodecyl sulfate/polyacrylamide gel electrophoresis (SDS-PAGE) following standard methods. Non-specific binding was blocked with 5% non-fat milk (dissolved in PBS) for 1h and then blots were incubated overnight at 4°C with antibodies against rabbit anti-mouse IL-2 (1∶500), rabbit anti-mouse IL-6 (1∶500), rabbit anti-mouse TNF-α (1∶500), mouse anti-mouse β-actin (1∶1000), and rabbit anti-mouse HMGB1 (1∶1000) diluted in 5% milk. β-actin was used as the internal reference for cytoplasmic proteins. All membranes were washed with PBST (1%Tween) and then incubated with a secondary goat anti-mouse or anti-rabbit antibody (1∶1000), dissolved in PBST, for 45 min at 37°C. Finally, membranes were washed with PBST three times for 5 min each and proteins were detected using the Odyssey two-color infrared laser imaging system (detected with fluorescence).

### 2.10 Total RNA Isolation and Real-time Reverse-transcriptase Polymerase Chain Reaction (RT-PCR)

mRNA transcripts were detected and analyzed via quantitative RT-PCR of the liver tissue. Total RNA was extracted from frozen liver tissue using TRIzol reagent (TIANGEN Biotech, China) as described by the manufacturer. To determine the expression of target genes in the liver, SYBR Green quantitative RT-PCR was performed using a 7900HT Fast Real-time PCR system (ABI, CA, USA) according to the instructions of SYBR Premix EX Taq (TaKaRa Biotechnology China). Primer sequences were as follows (shown in Table 1).

**Table 1:** 

Gene		Primer Sequence(5′→3′)
**TNF-α**	Forward	CAGGCGGTGCCTATGTCTC
	Reverse	CGATCACCCCGAAGTTCAGTAG
**IL-2**	Forward	TGAGCAGGATGGAGAATTACAGG
	Reverse	GTCCAAGTTCATCTTCTAGGCAC
**IL-6**	Forward	CTGCAAGAGACTTCCATCCAG
	Reverse	AGTGGTATAGACAGGTCTGTTGG
**HMGB1**	Forward	GCATCCTGGCTTATCCATTGG
	Reverse	GGCTGCTTGTCATCTGCTG
**NF-κB**	Forward	ATGGCAGACGATGATCCCTAC
	Reverse	CGGATCGAAATCCCCTCTGTT
**β-actin**	Forward	GGCTGTATTCCCCTCCATCG
	Reverse	CCAGTTGGTAACAATGCCATGT

### 2.11 Statistical Analysis

All results are expressed as the mean ± SD. The data of Real-time PCR and ELISA were analyzed using one-way analysis of variance (ANOVA). The results of ALT, AST, necrotic area, Western blot and Immunohistochemistry were analyzed using a Student’s t test. In all comparisons, *p*<0.05 were considered statistically significant. All statistical analyses were performed using SPSS 17.0 for Windows.

## Results

### 3.1 Preliminary Study Results

**Figure 1:**
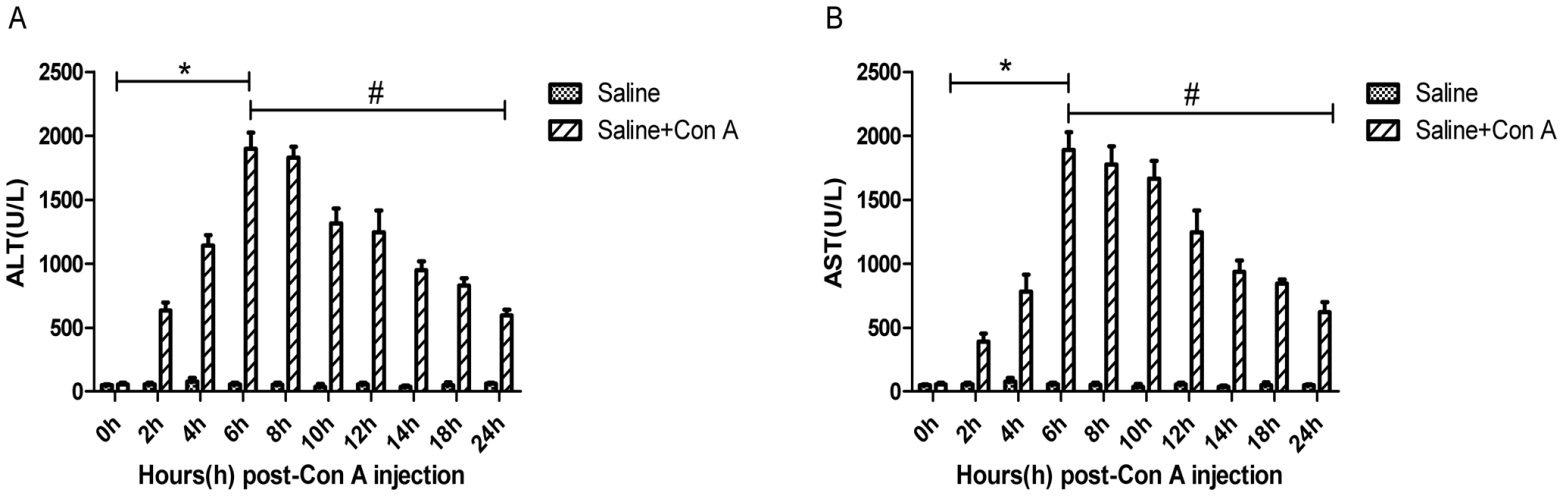
Preliminary study results. Mice (n = 5 for each group) were injected with Con A (20 mg/kg) at 0h. And every group were sacrificed at time point of 0h, 2h, 4h, 6h, 8h, 10h, 12h, 14h, 18h and 24h after the injection of Con A. A, B separately show the plasma ALT and AST levels at each time point. Data are expressed as mean ± SD (n = 5, *p<0.05 for Con A/0h VS Con A/6h, ^#^ *p*<0.05 for Con A/6h VS Con A/24h). Data analysis was performed by a student’s test.

**Figure 2:**
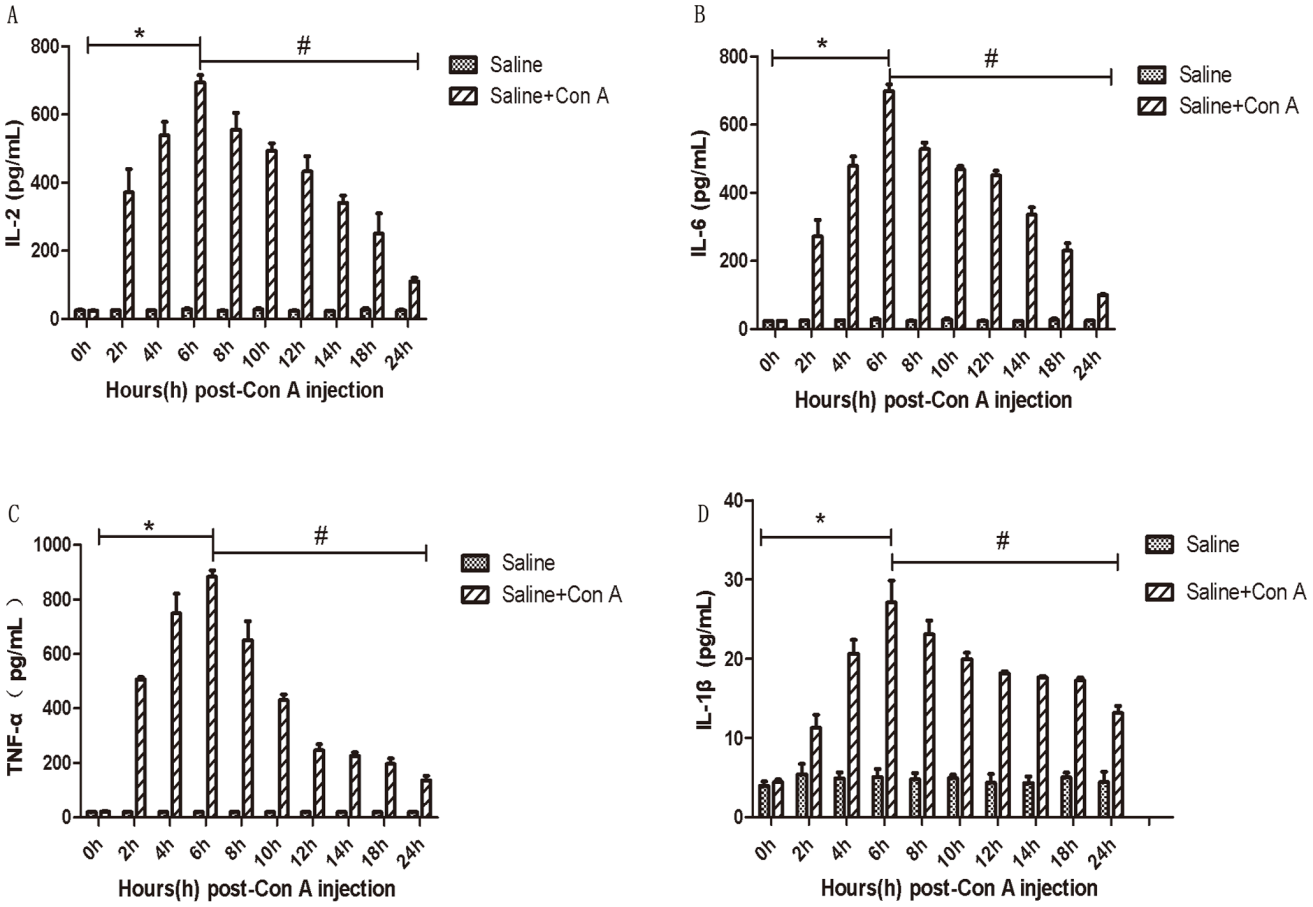
Preliminary study results. Mice (n = 5 for each group) were injected with Con A (20 mg/kg) at 0h. And every group were sacrificed at time point of 0h, 2h, 4h, 6h, 8h, 10h, 12h, 14h, 18h and 24h after the injection of Con A. A, B, C and D separately show the level of IL-2, IL-6, TNF-α and IL-1β in serum at each time point. Data are expressed as mean ± SD (n = 5, *p<0.05 for Con A/0h VS Con A/6h, ^#^ *p*<0.05 for Con A/6h VS Con A/24h).

The plasma ALT and AST levels from the first set of experiment are shown in Fig. 1. The levels of ALT and AST increased from 2h and reached peak levels at 6h, then decreased until 24h. The levels of IL-2, IL-6, TNF-α and IL-1β showed similar trends as seen in Fig. 2; a significant change can be seen between 0h, 6h and 24h. Therefore, we used the time points 3h, 6h and 24h in the second set of experiments.

### 3.2 Ethyl Pyruvate Pretreatment Attenuates Con A-induced Liver Injury in Mice

**Figure 3:**
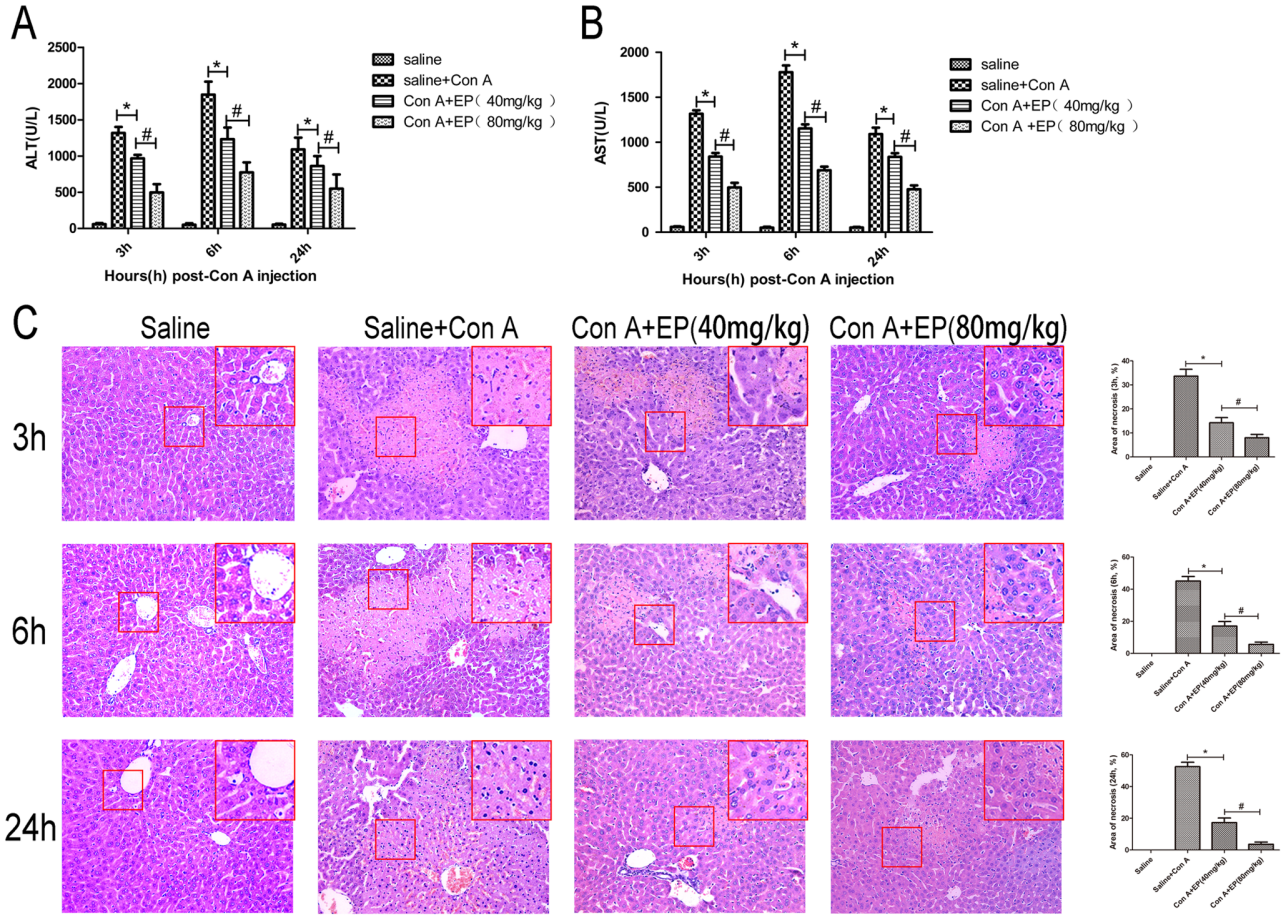
Ethyl pyruvate pretreatment attenuate Con A-induced autoimmune hepatitis. A, B. Effects of ethyl pyruvate on plasma ALT and AST levels at 3h, 6h, 24h after Con A injection in mice. Data are expressed as mean ± SD [n = 6, *p<0.05 for Saline+Con A vs. Con A+EP(40 mg/kg), ^#^ *p*<0.05 for Con A+EP(40 mg/kg) vs. Con A+EP(80 mg/kg) ]. C. Hematoxylin and eosin staining of liver sections (Saline or Saline+Con A or Con A+EP groups at the time point of 3h, 6h and 24h). Original magnification: ×200 and ×400. The necrotic areas were analyzed with Image-pro Plus 6.0, indicating there existed statistical significant among different groups. [n = 6, *p<0.05 for Saline+Con A vs. Con A+EP(40 mg/kg), ^#^ *p*<0.05 for Con A+EP(40 mg/kg) vs. Con A+EP(80 mg/kg)].

It is known that Con A can induce liver injury in mice, imitating T-cell mediated liver disease, including autoimmune hepatitis. To determine the effect of ethyl pyruvate on Con A-induced hepatitis, mice were treated with ethyl pyruvate 1h before Con A was administrated. Serum and liver tissue were collected at 3h, 6h and 24h according to the experimental design. The level of ALT and AST in serum was determined as shown in Fig. 3A and Fig. 3B; ALT and AST levels were significantly increased at the three time points. However, the elevation of ALT and AST was clearly decreased with ethyl pyruvate pretreatment. This same result was demonstrated in the histopathological study. As shown in Fig. 3C, we found massive areas of necrosis in the Con A-induced group. In contrast, the ethyl pyruvate-treated group showed minor liver damage, indicating ethyl pyruvate pretreatment significantly reduced liver necrosis. Ethyl pyruvate administered at 80 mg/kg was more effective. According to the results analyzed with Image-pro Plus 6.0, it is obvious to find there exist statistical significantamong different groups. These results show that ethyl pyruvate pretreatment attenuates Con A-induced autoimmune hepatitis in mice.

### 3.3 Effect of Ethyl Pyruvate on Production of IL-2, IL-6, IL-1β and TNF-α in Con-A Induced Hepatitis

**Figure 4:**
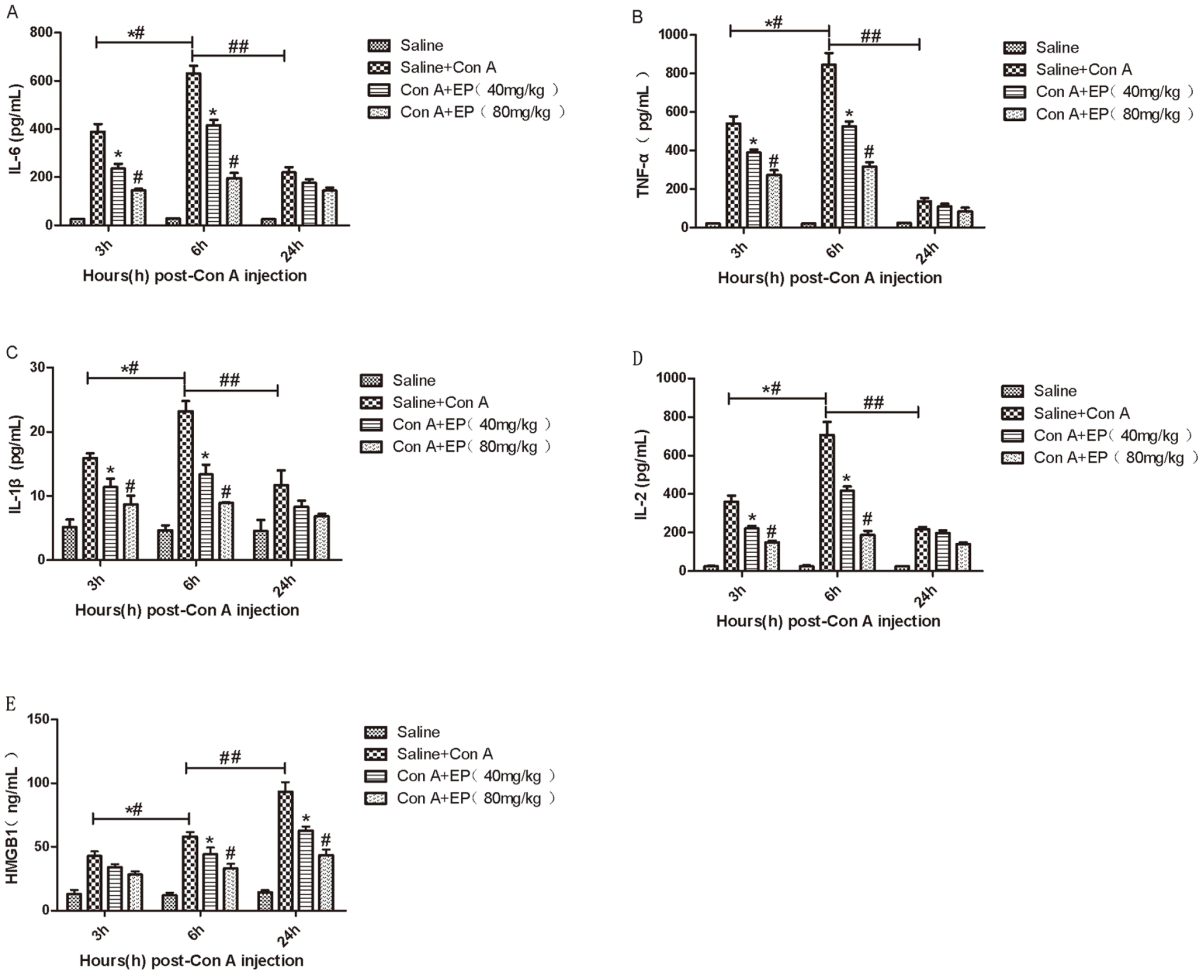
Effect of ethyl private on the release of IL-2, IL-6, TNF-α, IL-1β and HMGB1 in Con A-treated mice. IL-2, IL-6, TNF-α and IL-1β express maximally at 6h and HMGB1 expresses maximally at 24h. Ethyl pyruvate decreases the release of IL-2, IL-6, TNF-α and IL-1β at 3h and 6h and the release of HMGB1 at 6h and 24h after Con A injection. Data are showed as mean ± SD [n = 6, *p<0.05 for Con A+EP(80 mg/kg) vs. Saline+Con A; ^#^ *p*<0.05 for Con A+EP(40 mg/kg) vs. Con A+EP(80 mg/kg); *^#^ *p*<0.05 for Saline+Con A at 3h vs Saline+Con A at 6h; ^##^ *p*<0.05 for Saline+Con A/6h vs Saline+Con A/24h].

**Figure 5:**
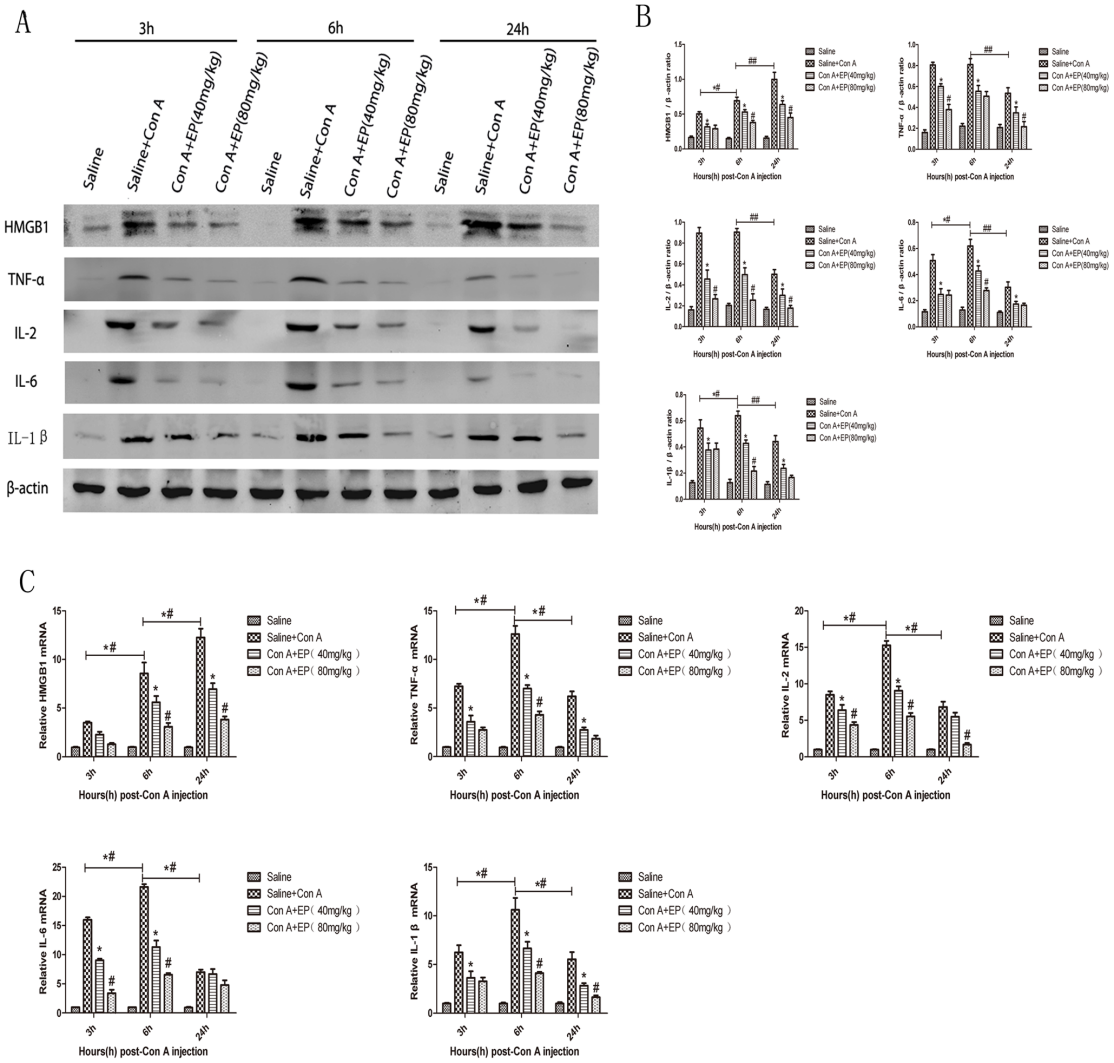
Ethyl pyruvate decreases the expression of IL-2, IL-6, TNF-α, IL-1β and HMGB1. A. Ethyl pyruvate inhibits the protein level of IL-2, IL-6, TNF-α, IL-1β and HMGB1 at all three time points detected by Western blot. HMGB1 significantly increased over time and other cytokines (IL-2, IL-6, TNF-α, IL-1β) expressed most at 6h. B. The results of western blot were analyzed with Quantity one. [n = 3, *p<0.05 for Con A+EP(80 mg/kg) vs. Saline+Con A; ^#^ *p*<0.05 for Con A+EP(40 mg/kg) vs. Con A+EP(80 mg/kg); *^#^ *p*<0.05 for Saline+Con A at 3h vs Saline+Con A at 6h; ^##^ *p*<0.05 for Saline+Con A/6h vs Saline+Con A/24h]. C. The mRNA expression of IL-2, IL-6, TNF-α, IL-1β and HMGB1 were evaluated by Real time PCR. Data are showed as mean ± SD[n = 3, *p<0.05 for Con A+EP(80 mg/kg) vs. Saline+Con A; ^#^ *p*<0.05 for Con A+EP(40 mg/kg) vs. Con A+EP(80 mg/kg); *^#^ *p*<0.05 for Saline+Con A at 3h vs Saline+Con A at 6h; ^##^ *p*<0.05 for Saline+Con A/6h vs Saline+Con A/24h].

It is a common perspective that the progress of hepatitis is associated with a series of proinflammatory cytokines such as IL-2, IL-6, IL-1β and TNF-α. Therefore, the levels of IL-2, IL-6, IL-1β and TNF-α in serum were determined by ELISA after Con A treatment, as shown in Fig. 4 (A, B, C, D). The levels of these cytokines increased after Con A induction and expressed most at 6h, and as expected, the production of IL-2, IL-6, IL-1β and TNF-α were prevented with ethyl pyruvate pretreatment, as seen at 3h and 6h. Furthermore, to confirm our observations, mRNA expression of IL-2, IL-6, IL-1β and TNF-α were detected by real-time PCR at the designed time points. Results (Fig. 5C) showed that mRNA expression of IL-2, IL-6, IL-1β and TNF-α were significantly increased in the Con A-treated group and ethyl pyruvate pretreatment diminished the mRNA expression of IL-2, IL-1β and TNF-α at all three time points and the expression of IL-6 obviously at 3h and 6h. In addition, IL-2, IL-6, IL-1β and TNF-α were maximally expressed at 6h, indicating these four cytokines mainly expressed in the early phase of Con A-induced hepatitis. Finally, the protein level of IL-2, IL-6, IL-1β and TNF-α were also assayed by western blot (Fig. 5A), where we observed that the expression of these cytokines decreased in the ethyl pyruvate treatment group compared to the Con A-induced group. And the protein level of IL-2, IL-6, IL-1β and TNF-α reached peak at 6h, similar to the mRNA expression (Fig. 5C). These results were analyzed with Quantity One, indicating there existed statistical significance among these changes (Fig. 5B). Thus, these results confirm that ethyl pyruvate pretreatment inhibits the production of proinflammatory cytokines, such as IL-2, IL-6, IL-1β and TNF-α in the early phase of Con A-induced hepatitis, which is associated with preventing Con A-induced hepatitis.

### 3.4 Effect of Ethyl Pyruvate on the Expression of HMGB1 in Con A-induced Hepatitis in Mice

We have shown that ethyl pyruvate is able to prevent Con A-induced hepatitis by down-regulating the production of inflammatory cytokines such as IL-2, IL-6, IL-1β and TNF-α. Here, we further explored the possible mechanism of ethyl pyruvate attenuation of Con A-induced hepatitis. It has been reported by Gong [18] that HMGB1 can exacerbate Con A-induced hepatitis. Therefore, we asked whether ethyl pyruvate acts by inhibiting HMGB1 in ConA-induced hepatitis. In one hand, the level of HMGB1 in serum was detected by ELISA, result showed that the expression of HMGB1 was most at 24h, and ethyl pyruvate could successfully inhibit it at 6h and 24h (Fig. 4E). On the other hand, the expression of HMGB1 mRNA was detected by real-time PCR, as shown in Fig. 5C. The level of HMGB1 mRNA was significantly upregulated in the Con A-induced group and downregulated after ethyl pyruvate treatment. This result was also observed for protein expression of HMGB1, as determined by western blot. As shown in Fig. 5A, HMGB1 was expressed at all three time points, with higher expression at 24h than at 3h and 6h. As expected, all doses of ethyl pyruvate in the pretreatment group resulted in lower expression of HMGB1 compared to the Con A-induced group. This result was analyzed with Quantity One and agreed with the change in HMGB1 mRNA expression we observed. Based on these results, we conclude that ethyl pyruvate pretreatment may ameliorate ConA-induced hepatitis partly through downregulation of HMGB1.

### 3.5 Effect of Ethyl Pyruvate on HMGB1 Activation in Con A-induced Hepatitis

**Figure 6:**
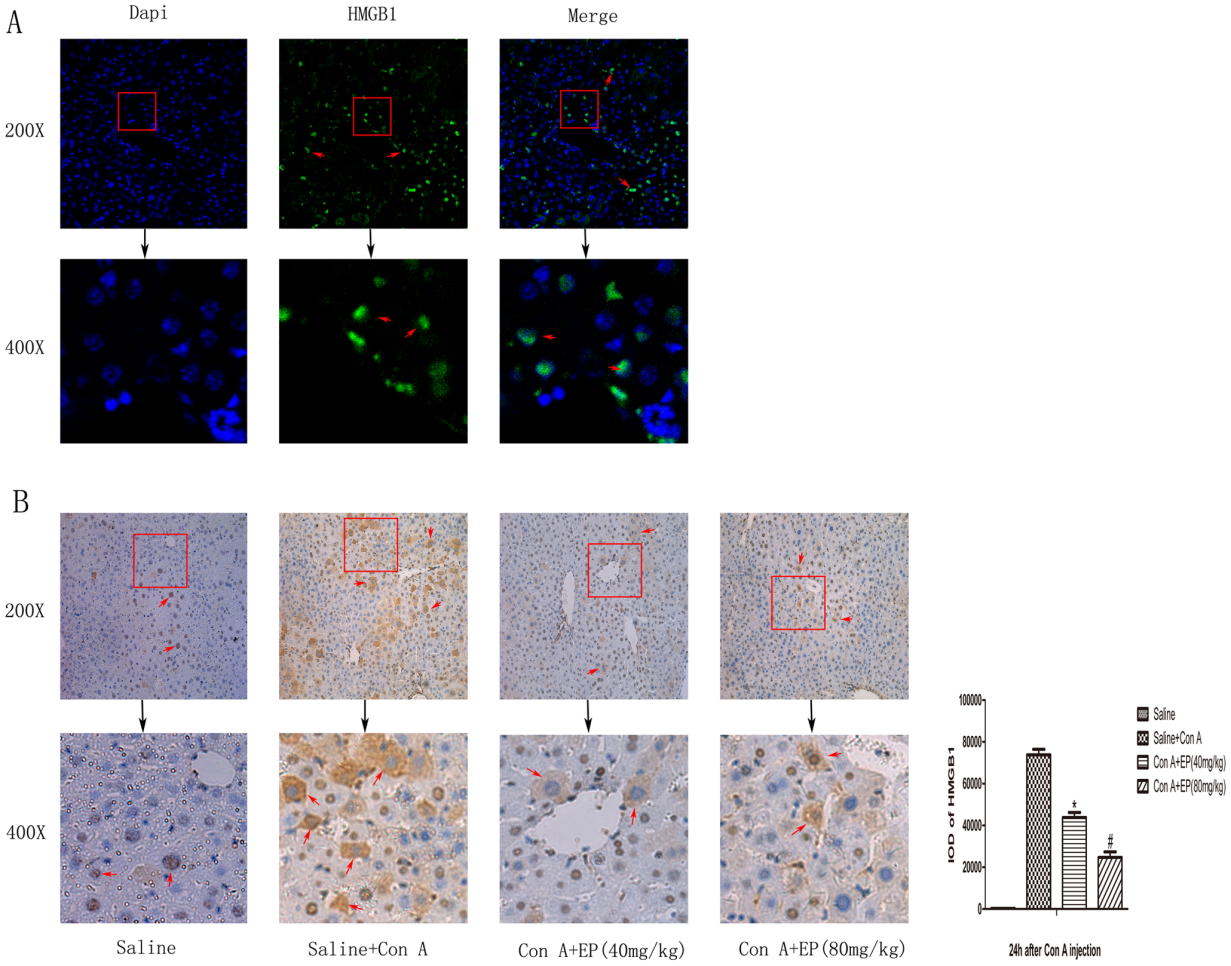
Ethyl pyruvate pretreatment significantly downregulated HMGB1 expression and translocation in Con A-induced mice. A. Location of HMGB1 in normal liver tissue collected at 24h was detected by immunofluoresence (original magnification: ×200 and ×400). Results showed HMGB1 expressed in nuclei indicated with red arrows. B. Liver tissues were collected 24h after Con A injection for immunohistochemical staining with anti-HMGB1 antibody (original magnification: ×200 and ×400). The IOD of HMGB1 in cytoplasm were analyzed by Image-Pro Plus 6.0. Results showed that ethyl pyruvate inhibited the expression and translocation of HMGB1 and had statistical significant. Data are showed as mean ± SD [n = 3, *p<0.05 for Saline+Con A vs. Con A+EP(40 mg/kg), ^#^ *p*<0.05 for Con A+EP(40 mg/kg) vs. Con A+EP(80 mg/kg) ]. The representative positive cells were indicated with red arrows.

HMGB1 plays a key role in the progression of inflammation as a lateproinflammatory cytokine. We observed HMGB1 mainly in the nucleus of normal liver tissue, as showed in Fig. 6A. Upon stimulation of the inflammatory signal, HMGB1 migrated from the nucleus to the cytoplasm to promote further inflammation. This transition was clearly shown by immunochemistry, as seen in Fig. 6B. In the normal control group, HMGB1 is mainly located in the nucleus and is hardly detected in the cytoplasm. As expected, HMGB1 was notably increased in the cytoplasm 24h after the injection of ConA partly in the area of necrosis, indicating that not only was HMGB1 released actively by inflammatory cells, but released passively due to injury and cell necrosis. Administration of ethyl pyruvate blocked the synthesis and migration of HMGB1, resulting in a significant decrease in the intensity of signal in the cytoplasm at 24h compared to the ConA-induced group. This result was analyzed with Image-pro Plus 6.0.

### 3.6 Effect of Ethyl Pyruvate on NF-κB Signal Pathway in Con A-induced Hepatitis in Mice

**Figure 7:**
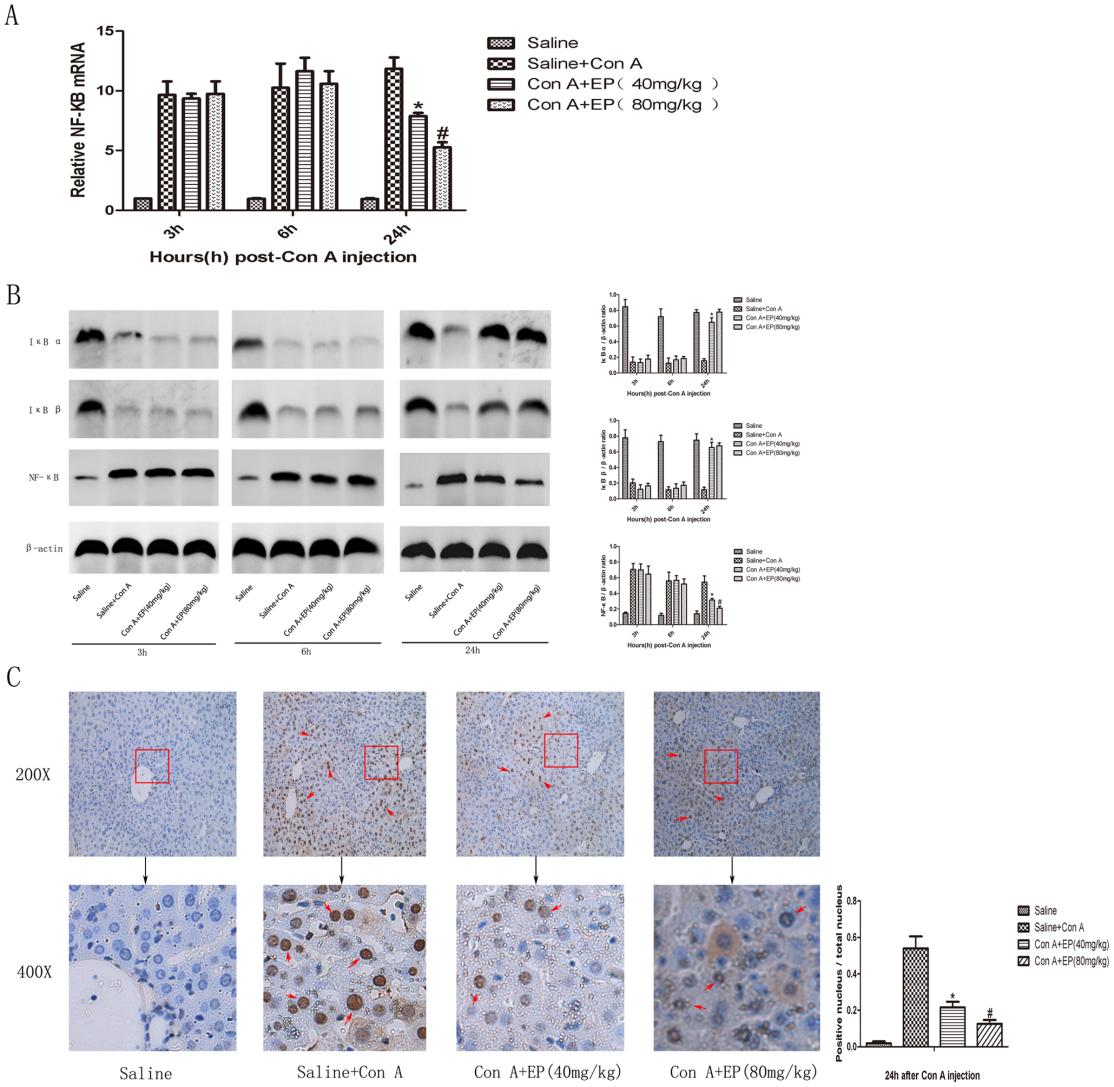
Ethyl pyruvate down-regulated NF-κB signal pathway at 24h. A. Real-time PCR was performed to detect mRNA expression of NF-κB. Compared to the Saline group, the expression of NF-κB in EP treatment group (40 mg/kg) is significantly decreased (*p<0.05), and the dose of 80 mg/kg has a more obviously effect (^#^ *p*<0.05). B. The expression of IκB α, IκB β and NF-κB was detected by western blot. The results were analyzed using Quantity One. [n = 3, *p<0.05 for Saline+Con A vs. Con A+EP(40 mg/kg), ^#^ *p*<0.05 for Con A+EP(40 mg/kg) vs. Con A+EP(80 mg/kg)] C. The expression level of NF-κB at 24h was detected by Immunohistochemistry with anti-NF-κB antibody. Original magnification: ×200 and ×400. The positive cells were indicated with red arrows. The result was analyzed using Image-pro Plus 6.0. [n = 3, *p<0.05 for Saline+Con A vs. Con A+EP(40 mg/kg), ^#^ *p*<0.05 for Con A+EP(40 mg/kg) vs. Con A+EP(80 mg/kg)].

NF-κB activation plays a key role in the induction of several proinflammatory mediators. To determine whether ethyl pyruvate down-regulated NF-κB signal pathway during Con A-induced hepatitis in mice, we firstly detected the protein level of IκB α and IκB β with western blot analysis. Results showed the degradation of IκB α and IκB β was obviously blocked by ethyl pyruvate at 24h, not at 3h and 6h (Fig. 7B). Furthermore, we explored the change of NF-κB in our model. We found that ethyl pyruvate significantly decreased the expression of NF-κB both in mRNA and protein level (Fig. 7A, Fig. 7B). We used Quantity One to analyze the result of western blot and demonstrated that these changes had statistical significant. Immunohistochemistry for NF-κB was detected to differentiate the location of NF-κB in different groups. As shown in Fig. 7C, we found that NF-κB mostly expressed and located in nuclei in Con A-induced group compared to Saline group. And the expression of NF-κB in nuclei was obviously decreased after ethyl pyruvate treatment at 24h. This result was analyzed with Image-pro Plus 6.0.

## Discussion

Liver disease is really a series of common and detrimental diseases such as acute hepatitis, chronic hepatitis, liver cirrhosis and hepatocellular carcinoma; there is a relationship among these diseases. Beasley et al. [27] reported that the incidence of primary hepatocellular carcinoma among carriers of hepatitis B surface antigen was much higher than among non-carriers in a prospective general population study of 22,707 Chinese men in Taiwan. A large amount of evidence has been accumulated confirming the relationship between hepatitis and hepatocellular carcinoma. More effective drugs are urgently needed for intervention at the hepatitis stage to decrease the incidence of hepatocellular carcinoma. Ethyl pyruvate, as a stable lipophilic ester derivative of pyruvate, has potential health benefits for humans and has attracted the attention of scientists. In this report, we demonstrated the effect of ethyl pyruvate on Con A-induced autoimmune hepatitis in mice.

Con A has the ability to activate T lymphocytes in vitro and cause T cell–dependent autoimmunehepatitis, and T lymphocytes are important contributors to the pathological process of hepatitis [1]. Recently, studies have reported that Con A-induced autoimmune hepatitis is largely mediated by the release of inflammatory cytokines such as IL-2, IL-4, IL-6, IL-10, IL-12, TNF-α and IFN-γ [28]. The timing of expression of these inflammatory cytokines plays an important role in the development of inflammation. IL-2, which has multiple effects on the immune system, is mainly released by activated T cells and participates in mediating inflammation and hemopoiesis. Sass et al. [28] explored the changes in cytokine expression in three mouse models of experimental hepatitisand found that the levels of IL-2 and IL-6 increased from 3h after the administration of Con A. It has also been reported that various concentrations of IL-2 played different roles in Con A-induced autoimmune hepatitis; a low dose of IL-2 exacerbated Con A-induced liver injury as reported by Zhang et al. [29]. In addition, TNF-α plays a pivotal role in inflammatory diseases and has been identified as a critical mediator in the experimental T cell-dependent disease models. It has been demonstrated that pretreatment of mice by polyclonal sheep anti-mouse TNF-α antiserum or a TNF-α inhibitor protected against Con A-induced liver injury [30]. Furthermore, mice deficient in the TNF-α related receptors TNFR1 and TNFR2 do not develop severe Con A-induced autoimmune hepatitis, as reported by Wolf et al. [31]. IL-2, IL-6, TNF-α and IL-1β were released starting at 3h after administration of Con A, and inhibition of the release of these cytokines attenuates the Con A-induced autoimmune hepatitis. In our studies, we showed that the level of IL-2, IL-6, TNF-α and IL-1β increased at 3h, reached peak levels at 6h, then diminished. We then showed that ethyl pyruvate pretreatment reduced the levels of IL-2, IL-6, TNF-α and IL-1β at 3h and 6h in Con A treated mice; the corresponding pathological features were also ameliorated in the ethyl pyruvate-treated group at 3h and 6h. Therefore, we consider that ethyl pyruvate may ameliorate Con A-induced liver injury through the reduction of inflammatory cytokines such as IL-2, IL-6, TNF-α and IL-1β.

HMGB1, a highly conserved nuclear protein, not only mediates gene transcription and maintains the stability of the nucleosome structure, it is also regarded as a central mediator of inflammation. Andersson et al. [32] reported that recombinant HMGB1 could upregulate TNF mRNA and protein expression in human blood mononuclear cell cultures and, in vivo, the expression of HMGB1 increased in experimental severe acute pancreatitis, as shown by Yasuda et al. [33]. HMGB1 has also been studied in the clinic. Huang et al. [34] found plasma HMGB1 levels increased significantly in 338 patients of ischemic stroke and a similar study was also reported in clinical acute lung injury [12]. The release of HMGB1 is delayed relative to classical early cytokines such as TNF-α, IL-6, IL-2 and IL-1β. Here, we observed that the levels of TNF-α, IL-6, IL-2 and IL-1β were higher at 3h and 6h after Con A treatment than at 24h, both in serum and in tissue. On the other hand, the levels of HMGB1 increased gradually and reached peak levels at 24h post Con A treatment. This indicates that HMGB1 may serve a pivotal role in the later processes of Con A-induced hepatitis. When HMGB1 is activated, it migrates from the nucleus to the cytoplasm to increase inflammation. Here, we observed that HMGB1 expression was increased in the cytoplasm in Con A-induced mice. Because HMGB1 is involved in inflammation, HMGB1 is a potential target for inhibition of inflammation. Sawa et al. [35] reported that blockade of HMGB1 protein attenuated experimental severe acute pancreatitis. Recently, this result has been confirmed in animal models, such as traumatic brain injury, carotid artery injury, and liver injury in heatstroke [16]. Here, we explored whether ethyl pyruvate ameliorated inflammation by decreasing the expression of HMGB1 in Con A-induced autoimmune hepatitis in mice. We found that the level of HMGB1 in serum and tissue decreased significantly in the ethyl pyruvate pre-treatment group. It appears that ethyl pyruvate not only regulates the expression of newly synthesized HMGB1, but also suppresses HMGB1 transfer from the nucleus to the cytoplasm.

The mechanism behind how ethyl pyruvate decreases the expression of HMGB1 is not clear. It has recently been reported by Wang et al. [36] that an NF-κB inhibitor could inhibit HMGB1 expression in lung tissues of rats with COPD. Therefore, we firstly detected the expression of IκB α and IκB β in our model with western blot, results showed that the degradation of IκB α and IκB β was significantly blocked by ethyl pyruvate. Secondly, the expression of NF-κB was also decreased by ethyl pyruvate. Taking these results, it can be demonstrated that ethyl pyruvate has the ability to down-regulate NF-κB signal pathway. Therefore, it is possible that ethyl pyruvate may decrease the expression of HMGB1 by down-regulating NF-κB signal pathway. There is also cross-talk between HMGB1 and other proinflammatory cytokines, such as TNF-α and IL-6; it has been reported that HMGB1 upregulated the level of TNF-α and IL-6 functioning as a later proinflammatory cytokine [30]. However, to a certain extent, these data appear to be in accordance with our results. Collectively, these data imply that there are other mechanisms involved in mediating the effects of ethyl pyruvate on HMGB1 in Con A-induced autoimmune hepatitis. We will focus on exploring these unknown mechanisms in the future.

## Conclusions

The purpose of our study was to determine whether ethyl pyruvate could inhibit the expression and release of both early (TNF-α, IL-2, IL-6, IL-1β) and late (HMGB1 ) cytokines in Con A-induced autoimmune hepatitis. Our results are as follows: (1) ethyl pyruvate attenuate Con A-induced autoimmune hepatitis in Balb/C mice; (2) ethyl pyruvate decreases the TNF-α, IL-2, IL-6, IL-1β, and HMGB1 expression in vivo; (3) ethyl pyruvate may inhibit the release of HMGB1 through modulation of NF-κB signal pathway. Although there are further mechanisms to be explored, our study provides a new approach for the treatment of acute hepatitis in the clinic.

## References

[1] Tiegs G, Hentschel J, Wendel A (1992) A T cell-dependent experimental liver injury in mice inducible by concanavalin A. J Clin Invest. 90: 196–203. 10.1172/JCI115836 PMC443081 1634608

[2] Kusters S, Gantner F, Kunstle G, Tiegs G (1996) Interferon gamma plays a critical role in T cell-dependent liver injury in mice initiated by concanavalin A. Gastroenterology. 111: 462–471. 10.1053/gast.1996.v111.pm8690213 8690213

[3] Pisetsky DS (2013) The Translocation of Nuclear Molecules During Inflammation and Cell Death. Antioxid Redox Signal. 10.1089/ars.2012.5143 PMC3928723 23373769

[4] Tiegs G (2007) Cellular and cytokine-mediated mechanisms of inflammation and its modulation in immune-mediated liver injury. Z Gastroenterol 45: 63–70. 17236122 10.1055/s-2006-927397

[5] Schwabe RF, Brenner DA (2006) Mechanisms of Liver Injury. I. TNF-alpha-induced liver injury: role of IKK, JNK, and ROS pathways. Am J Physiol Gastrointest Liver Physiol 290: G583–589. 16537970 10.1152/ajpgi.00422.2005

[6] Zhou Y, Dai W, Lin C, Wang F, He L, et al. (2013) Protective effects of necrostain-1 against concanavalin A-induced acute hepatic injury in mice. Mediators Inflamm 706156. doi: 10.1155/2013/706156. Epub 2013 Oct 1. 10.1155/2013/706156 PMC3806455 24198446

[7] Tang D, Kang R, Zeh HJ 3rd, Lotze MT (2011) High-mobility group box 1, oxidative stress, and disease. Antioxid Redox Signal 14: 1315–1335. 20969478 10.1089/ars.2010.3356 PMC3048826

[8] Nogueira-Machado JA, de Oliveira Volpe CM (2012) HMGB-1 as a target for inflammation controlling. Recent Pat Endocr Metab Immune Drug Discov 6: 201–209. 22845335 10.2174/187221412802481784

[9] Wang H, Bloom O, Zhang M, Vishnubhakat JM, Ombrellino M, et al (1999) HMG-1 as a late mediator of endotoxin lethality in mice. Science 285: 248–251. 10398600 10.1126/science.285.5425.248

[10] Kim S, Kim SY, Pribis JP, Lotze M, Mollen KP, et al (2013) Signaling of high mobility group box 1 (HMGB1) through toll-like receptor 4 in macrophages requires CD14. Mol Med 19: 88–98. 23508573 10.2119/molmed.2012.00306 PMC3667211

[11] Rosas-Ballina M, Goldstein RS, Gallowitsch-Puerta M, Yang L, Valdes-Ferrer SI, et al (2009) The selective alpha7 agonist GTS-21 attenuates cytokine production in human whole blood and human monocytes activated by ligands for TLR2, TLR3, TLR4, TLR9, and RAGE. Mol Med 15: 195–202. 19593403 10.2119/molmed.2009.00039 PMC2707516

[12] Kang R, Tang D, Schapiro NE, Loux T, Livesey KM, et al. (2013) The HMGB1/RAGE inflammatory pathway promotes pancreatic tumor growth by regulating mitochondrial bioenergetics. Oncogene. 10.1038/onc.2012.631 PMC3795800 23318458

[13] Ueno H, Matsuda T, Hashimoto S, Amaya F, Kitamura Y, et al (2004) Contributions of high mobility group box protein in experimental and clinical acute lung injury. Am J Respir Crit Care Med 170: 1310–1316. 15374839 10.1164/rccm.200402-188OC

[14] Andrassy M, Volz HC, Schuessler A, Gitsioudis G, Hofmann N, et al (2012) HMGB1 is associated with atherosclerotic plaque composition and burden in patients with stable coronary artery disease. PLoS One 7: e52081. 23284878 10.1371/journal.pone.0052081 PMC3524090

[15] Zhou RR, Liu HB, Peng JP, Huang Y, Li N, et al (2012) High mobility group box chromosomal protein 1 in acute-on-chronic liver failure patients and mice with ConA-induced acute liver injury. Exp Mol Pathol 93: 213–219. 22609241 10.1016/j.yexmp.2012.05.006

[16] Tong H, Tang Y, Chen Y, Yuan F, Liu Z, et al (2013) HMGB1 activity inhibition alleviating liver injury in heatstroke. J Trauma Acute Care Surg 74: 801–807. 23425738 10.1097/TA.0b013e31827e9a65

[17] Watanabe T, Kubota S, Nagaya M, Ozaki S, Nagafuchi H, et al (2005) The role of HMGB-1 on the development of necrosis during hepatic ischemia and hepatic ischemia/reperfusion injury in mice. J Surg Res 124: 59–66. 15734480 10.1016/j.jss.2004.10.019

[18] Gong Q, Zhang H, Li JH, Duan LH, Zhong S, et al (2010) High-mobility group box 1 exacerbates concanavalin A-induced hepatic injury in mice. J Mol Med (Berl) 88: 1289–1298. 20848269 10.1007/s00109-010-0681-7

[19] Tang D, Kang R, Xiao W, Zhang H, Lotze MT, et al (2009) Quercetin prevents LPS-induced high-mobility group box 1 release and proinflammatory function. Am J Respir Cell Mol Biol 41: 651–660. 19265175 10.1165/rcmb.2008-0119OC PMC2784404

[20] Li W, Ashok M, Li J, Yang H, Sama AE, et al (2007) A major ingredient of green tea rescues mice from lethal sepsis partly by inhibiting HMGB1. PLoS One 2: e1153. 17987129 10.1371/journal.pone.0001153 PMC2048740

[21] Tu CT, Yao QY, Xu BL, Wang JY, Zhou CH, et al (2012) Protective effects of curcumin against hepatic fibrosis induced by carbon tetrachloride: modulation of high-mobility group box 1, Toll-like receptor 4 and 2 expression. Food Chem Toxicol 50: 3343–3351. 22683883 10.1016/j.fct.2012.05.050

[22] Fink MP (2007) Ethyl pyruvate: a novel anti-inflammatory agent. J Intern Med 261: 349–362. 17391109 10.1111/j.1365-2796.2007.01789.x

[23] Ulloa L, Ochani M, Yang H, Tanovic M, Halperin D, et al (2002) Ethyl pyruvate prevents lethality in mice with established lethal sepsis and systemic inflammation. Proc Natl Acad Sci U S A 99: 12351–12356. 12209006 10.1073/pnas.192222999 PMC129448

[24] Luan ZG, Zhang H, Ma XC, Zhang C, Guo RX (2012) Therapeutic treatment with ethyl pyruvate attenuates the severity of liver injury in rats with severe acute pancreatitis. Pancreas 41: 729–737. 22699144 10.1097/MPA.0b013e31823cd3ef

[25] Xu XH, Chen Q, Chen Y, Lv LX, Zhu CQ, et al (2010) [Effect of ethyl pyruvate on expression of inflammatory factors and mitogen-activated protein kinase proteins in renal ischemic/reperfusion injury in BABL/c mice]. Zhongguo Wei Zhong Bing Ji Jiu Yi Xue 22: 750–753. 21190605

[26] Wang LW, Wang LK, Chen H, Fan C, Li X, et al (2012) Ethyl pyruvate protects against experimental acute-on-chronic liver failure in rats. World J Gastroenterol 18: 5709–5718. 23155311 10.3748/wjg.v18.i40.5709 PMC3484339

[27] Beasley RP, Lin CC, Chien CS, Chen CJ, Hwang LY (1982) Geographic distribution of HBsAg carriers in China. Hepatology 2: 553–556. 6288542 10.1002/hep.1840020507

[28] Sass G, Heinlein S, Agli A, Bang R, Schumann J, et al (2002) Cytokine expression in three mouse models of experimental hepatitis. Cytokine 19: 115–120. 12242077 10.1006/cyto.2002.1948

[29] Zhang X, Wei HX, Rui S, Wei H, Tian Z (2010) Opposite effects of high and low doses of interleukin-2 on T cell-mediated hepatitis in mice (interleukin-2 on hepatitis). Hepatol Int 4: 641–648. 21063489 10.1007/s12072-010-9196-0 PMC2940003

[30] Nakamura K, Okada M, Yoneda M, Takamoto S, Nakade Y, et al (2001) Macrophage inflammatory protein-2 induced by TNF-alpha plays a pivotal role in concanavalin A-induced liver injury in mice. J Hepatol 35: 217–224. 11580144 10.1016/s0168-8278(01)00109-x

[31] Wolf D, Hallmann R, Sass G, Sixt M, Kusters S, et al (2001) TNF-alpha-induced expression of adhesion molecules in the liver is under the control of TNFR1–relevance for concanavalin A-induced hepatitis. J Immunol 166: 1300–1307. 11145713 10.4049/jimmunol.166.2.1300

[32] Andersson U, Wang H, Palmblad K, Aveberger AC, Bloom O, et al (2000) High mobility group 1 protein (HMG-1) stimulates proinflammatory cytokine synthesis in human monocytes. J Exp Med 192: 565–570. 10952726 10.1084/jem.192.4.565 PMC2193240

[33] Yasuda T, Ueda T, Shinzeki M, Sawa H, Nakajima T, et al (2007) Increase of high-mobility group box chromosomal protein 1 in blood and injured organs in experimental severe acute pancreatitis. Pancreas 34: 487–488. 17446855 10.1097/MPA.0b013e31804154e4

[34] Huang JM, Hu J, Chen N, Hu ML (2013) Relationship between plasma high-mobility group box-1 levels and clinical outcomes of ischemic stroke. J Crit Care 28: 792–797. 23137435 10.1016/j.jcrc.2012.10.003

[35] Sawa H, Ueda T, Takeyama Y, Yasuda T, Shinzeki M, et al (2006) Blockade of high mobility group box-1 protein attenuates experimental severe acute pancreatitis. World J Gastroenterol 12: 7666–7670. 17171797 10.3748/wjg.v12.i47.7666 PMC4088050

[36] Wang CM, Jiang M, Wang HJ (2013) Effect of NF-kappa B inhibitor on high-mobility group protein B 1 expression in a COPD rat model. Mol Med Res 7(2): 499–502. 10.3892/mmr.2012.1181 23151670

